# Guillain-Barre syndrome caused by hepatitis E infection: case report and literature review

**DOI:** 10.1186/s12879-018-2959-2

**Published:** 2018-01-23

**Authors:** Xiaoqin Zheng, Liang Yu, Qiaomai Xu, Silan Gu, Lingling Tang

**Affiliations:** Zhejiang University First Affiliated Hospital State Key Laboratory for Diagnosis and Treatment of Infectious Diseases, Hangzhou, Zhejiang China

**Keywords:** Hepatitis E infection, Guillain–Barre syndrome, Viral hepatitis, Extra-hepatic manifestations

## Abstract

**Background:**

Hepatitis E infection is a global disorder that causes substantial morbidity. Numerous neurologic illnesses, including Guillain–Barre syndrome (GBS), have occurred in patients with hepatitis E virus (HEV) infection.

**Case presentation:**

We report a 58 year-old non-immunocompromised man who presented with progressive muscle weakness in all extremities during an episode of acute HEV infection, which was confirmed by measuring the anti-HEV IgM antibodies in the serum. Both cerebrospinal fluid examination and electrophysiological study were in agreement with the diagnosis of HEV-associated GBS. Following the treatment with intravenous immunoglobulin, the patient’s neurological condition improved rapidly.

**Conclusions:**

HEV infection should be strongly considered in patients with neurological symptoms, especially those with elevated levels of liver enzymes.

**Electronic supplementary material:**

The online version of this article (10.1186/s12879-018-2959-2) contains supplementary material, which is available to authorized users.

## Background

Hepatitis E virus (HEV) infection, one of the most common causes of acute viral hepatitis, is an important public-health concern and leads to substantial morbidity [1]. HEV causes acute and chronic hepatitis, but most of these infections are asymptomatic [2]. In symptomatic patients, HEV can cause fulminant acute hepatitis, fibrosis, or cirrhosis [3, 4] Numerous extra-hepatic manifestations, including many neurological illnesses, are associated with acute or chronic hepatitis E [5]. In this article, we report a rare case of a patient clinically diagnosed with Guillain-Barre syndrome (GBS) due to acute HEV infection. We also reviewed English language scientific literature for the clinical characteristics of HEV associated with GBS.

## Case presentation

A 58 year-old non-immunocompromised man presented general fatigue, anorexia, cough, mild jaundice, and excretion of tea-colored urine for 7 days. He was referred to a local hospital, wherein his liver function tests showed elevated levels of aspartate aminotransferase (AST) at 273 U/L, alanine aminotransferase (ALT) at 664 U/L, total bilirubin at 51.8 μmol/L, and conjugated bilirubin at 20.5 μmol/L. Serological study was positive for IgM antibodies for HEV. A working diagnosis of acute hepatitis E was performed, and liver protection treatment was applied to the patient. However, on the 4th day of admission, the patient complained about progressive muscle weakness on his lower limbs, numbness, and abnormal pinprick sensation in his plantar that render him unable to walk. The patient was subsequently transferred to our hospital for further treatment because of the rapidly progressive symmetrical weakness of his lower and upper limbs.

The patient had a history of recovered schistosomiasis. He had never been exposed to a polluted environment or affected animals. He also had no history of blood transfusions, risky sexual behavior, or drug addiction.

Upon admission, general examination on the patient revealed unremarkable findings. His temperature was 36.4 °C, and his blood pressure was 130/99 mmHg. Physical examination showed asthenia, jaundice, blepharoptosis, and paresthesia. His cranial nerve examination showed unilateral facial nerve palsy, Romberg’s sign and the straight leg raising test was positive. Motor weakness was present in all limbs, with power of 4/5 in the upper limbs and 2/5 in the lower limbs. His triceps, biceps, and brachioradialis reflexes were normal, whereas his patellar and Achilles tendon reflexes were absent bilaterally. Two days after admission, he developed dysphagia, choking, areflexia and labored breathing. Neurological examination showed and unilateral cranial palsy with left blepharoptosis, flat nasolabial fold and incomplete eyelid closure of right side. Muscle weakness in his four limbs progressed rapidly. The power of the upper extremity was 2/5 for the proximal muscles and 4/5 for the distal muscles. The power grade of both proximal and distal legs was 1/5. GBS was suspected, and lumbar puncture was conducted on the 2nd day. Cerebrospinal fluid (CSF) examination showed 0/μL monocyte, 4.6 mmol/L glucose level, and 275.3 mg/dL protein level, which suggested albuminocytologic dissociation. Nerve conduction studies showed evidence of demyelinating neuropathy with dysfunction of motor and sensory nerve fibers.

Laboratory investigations showed 20 μmol/L total bilirubin, 10 μmol/L conjugated bilirubin, 126 U/L alanine aminotransferase, and 160 U/L gamma-glutamyl transpepidase. Serologic studies for IgM and IgG anti-HEV were both positive. No serological evidence was found for hepatitis A virus, hepatitis B virus, hepatitis C virus, hepatitis D virus, and syphilis or human immunodeficiency virus. Epstein–Barr virus and cytomegalovirus serology indicated positive IgG. Serum antibodies to anti-ganglioside antibodies GM1 and GM2 were negative, while the level of serum immunoglobulin G was increased.

**Fig. 1:**
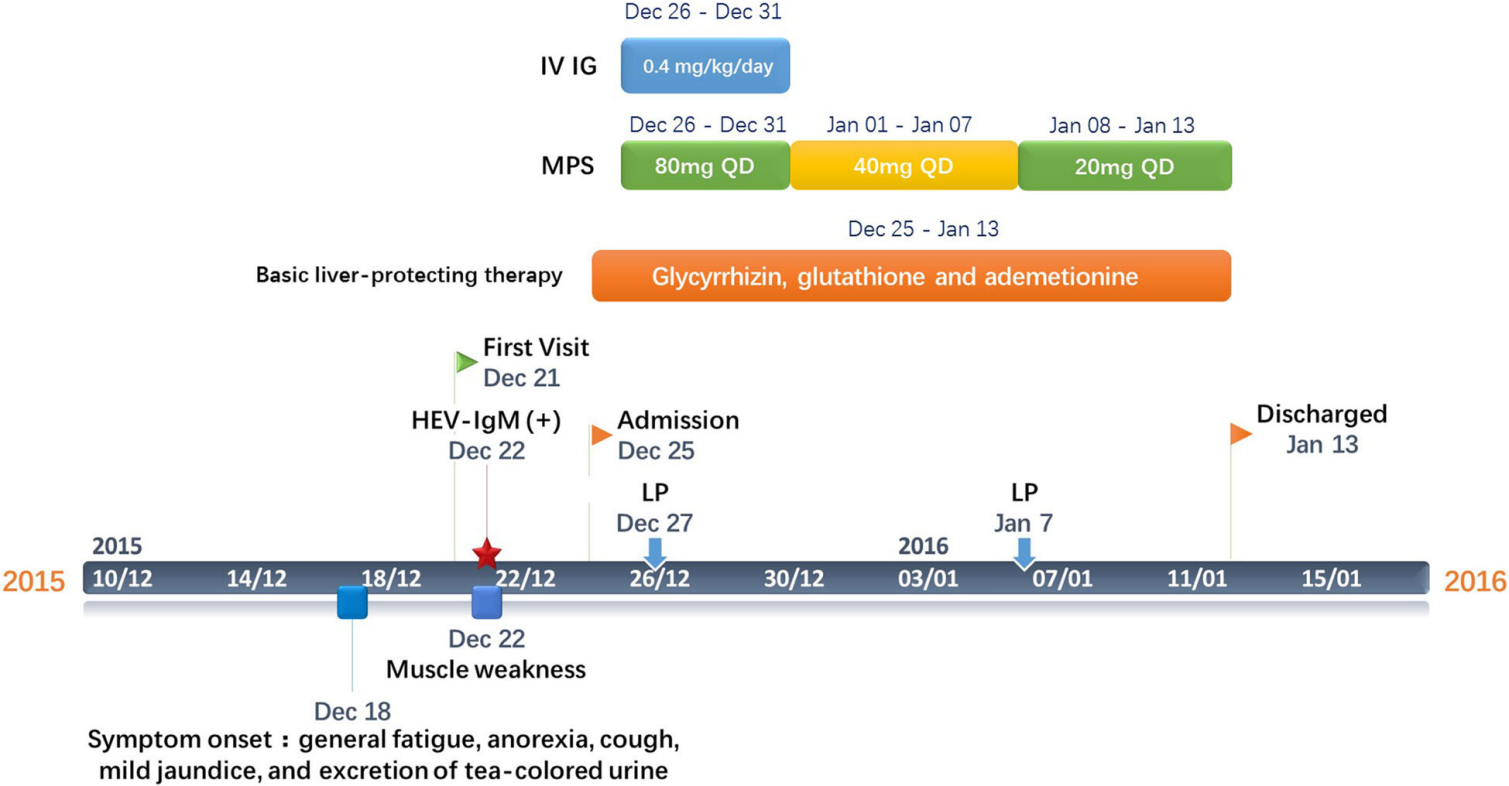
Medical History. IVIg: intravenous immunoglobulin; MPS: Methylprednisolone; LP: lumbar puncture

Cerebral computed tomography and magnetic resonance imaging scans indicated normal results. (The clinical course is summarized in Fig. 1).

The clinical history, physical examination, and biochemical findings of the patient were consistent with the diagnosis of acute HEV-associated GBS. He was treated with intravenous immunoglobulin at a dose of 0.4 mg/kg per day for 5 days. Meanwhile, methylprednisolone were also used to suppress inflammatory response. Glycyrrhizin, glutathione, and ademetionine were simultaneously administered for liver therapy. During the next 2 weeks, his clinical condition and muscle power improved gradually, and the patient had no complaints of respiratory distress or malaise. Repeat CSF examination 2 weeks after admission revealed 10/μL monocyte and 85.7 mg/dL protein level. At discharge, the patient had 5/5 power bilaterally in his arms and 4/5 power bilaterally in his legs. A month later, his liver function substantially improved, and his serum levels of AST and ALT were nearly normal. Six months after discharge, serological study (Wantai HEV-IgM ELISA) showed that IgM anti-HEV antibodies became negative, which suggested full recovery from the acute phase of hepatitis E. The patient responded well to treatment with his muscle power returning to normal, but still felt weakness in his right arm.

## Discussion and conclusions

HEV, previously known as waterborne or enterically transmitted viral hepatitis, is hyper-endemic in many developing countries and endemic in developed countries [6]. Aside from hepatitis symptoms, HEV-associated neurological injuries also cause significant morbidity. Neurologic complications develop in 7 (5.5%) out of 126 patients with acute and chronic HEV infections in the United Kingdom and France [7]. The clinical spectrum of neurological injury is broad. GBS and neuralgic amyotrophy are the most frequently reported conditions [8]. Other neurological disorders include transverse myelitis, encephalitis, cranial nerve palsy, and meningoradiculitis [9, 10].

GBS is an acute immune-mediated polyradiculoneuropathy that results in rapidly progressing symmetric motor paralysis, limb palsy, hypoflexia, areflexia, and other neurological disorders. GBS is also a heterogeneous disorder with several forms, including acute inflammatory demyelinating polyneuropathy (AIDP), acute motor axonal neuropathy (AMAN), acute motor–sensory axonal neuropathy (AMSAN), and Miller Fisher syndrome (MFS). GBS is usually preceded by an infection, which evokes an immune response that cross-reacts with peripheral nerve components via molecular mimicry [11]. Anti-ganglioside GM1or GM2 positive has been measured in three out of six cases of HEV-associated GBS. This condition may lead to autoimmune inflammatory polyneuropathy [12–17].

A literature review was performed using the PubMed database to identify other published cases and clarify the clinical characteristics of HEV-associated GBS. We used combinations of keywords, including hepatitis E and Guillain–Barre syndrome, and hepatitis E and MFS. Fifty-two cases described HEV-associated GBS. With the addition of our patient, 53 cases were counted, and the clinical characteristics of these cases are summarized in Table 1. The mean age of the reported patients was 47 years (20–73 years), and most of them are middle-aged men (30 men out of 53 cases: 57.7%). These patients developed HEV-associated GBS within an acute onset and have experienced hepatitis symptoms, including jaundice, malaise, nausea, and vomiting. GBS symptoms include motor weakness, cranial nerve palsy, and sensory disorder, which usually manifest after the occurrence of hepatitis symptoms. The mean delay between acute hepatitis E and GBS symptoms was 12 days (with a range of 3–73). Genotyping was performed among 11patients. Most of these patients revealed type 3, which suggested that HEV3 had high tropism of GBS. Most of our reviewed cases were found in Western Europe and Southern and Eastern Asia, where genotype 3 is prevalent. HEV RNA was found in the serum of 17 patients. Furthermore, HEV RNA was positive in the serum and the CSF of one patient.

**Table 1:** Clinical characteristics of HEV-associated GBS

Reference	Country	No. of cases	Age	Sex	Delay hepatitis neurological manifestation	Nerve conduction study	Anti-glycoprotein antibody	IgM	HEV RNA	HEV genotype	ALT (IU/L)		CSF		Treatment	Recovery/Delay	Comorbidity
												Bilirubin(μmol/L)	Protein level, mg/dL	Cell count (×10^6/L)			
Sood 2000 [13]	India	1	50	M	5 days	AIDP	NT	+	NT	NT	114	242.82	186	0	Supportive	Full/1 month	None
Kumar 2002 [16]	India	1	35	M	17 days	AMSAN	NT	+	NT	NT	752	91.8	NT	NT	MV/IVIg	Full/2 weeks	None
Kamani 2005 [12]	India	1	58	F	9 days	NT	NT	+	NT	NT	1448	40.7	80	2	IVIg/PP	Full/12 days	None
Khanam 2008 [19]	Bangladesh	1	20	M	10 days	AIDP&AMSAN	NT	+	NT	NT	2509	61.56	190	0	MV	Full/12 days	None
Loly 2009 [14]	Belgium	1	66	M	Few days	AIDP	GM2+	+	NT	NT	1813	NM	172.2	NM	IVIg	Full/4 months	None
Cronin 2011 [15]	Ireland	1	40	M	Concomitant	AIDP	GM2+	+	NT	NT	57	NM	>500	0	MV/IVIg/PP	Full/6 months	None
Kamar 2011 [7]	France	1	60	F	Concomitant	AIDP	NT	+	Serum+ CSF-	3f	384	35	200	14	IVIg	Partial/18 months	None
Maurissen 2012 [17]	Belgium	1	51	F	Concomitant	AIDP	GM1&GM2+	+	Serum+	NT	2704	NM	61	15	IVIg	Full/1 week	None
Del Bello 2012 [20]	France	1	65	M	Concomitant	AIDP	NT	+	Serum+	3f	2000	NM	64	0	MV/IVIg/Ribavirin	partial/2 month	Severe myositis
Tse 2012 [21]	Hong Kong	1	60	F	3 days	AIDP	NT	+	NT	NT	2858	60	NM	NM	PP	Full/1 month	None
Santos 2013 [22]	Portugal	1	58	M	17 days	AIDP	NT	+	Serum+	3a	2320	114.74	181	4	MV/IVIg	partial/2 month	None
Sharma 2013 [23]	India	1	27	M	40 days	AIDP	NT	+	NT	NT	NM	NM	243	<5	IVIg	Full/NM	None
Geurtsvan-Kessel 2013 [24]	Bangladesh	11	mean: 24	NM	NM	NM	NT	+	Serum+(*n* = 1)	1(*n* = 1)	NM	NM	NM	NM	NM	NM	None
van den Berg 2014 [25]	Netherlands	10	mean: 54	6 M, 4F	Mean: 5 days	AIDP(*n* = 5) AMAN(*n* = 1) AMSAN(*n* = 1) Equivocal(*n* = 2) Inexcitable(*n* = 1)	NT	+	Serum+(*n* = 3) CSF-(*n* = 10)	3	Mean: 160.9	Mean: 13.3	NM	Mean:4.2	NM	NM	NM
Chen 2014 [26]	China	1	64	M	5 days	AIDP	GM2+	+	NT	NT	1461	156.9	88	10	MV/IVIg	Full/12 months	Encephalitis
Scharn 2014 [27]	Germany	1	50	M	7 days	AIDP & AMAN	–	+	Serum+ CSF-	3c	334	NM	81	<1	IVIg	Partial/5 months	None
Woolson 2014 [28]	NM	1	42	M	NM	NM	NT	+	Serum+	3	623	16	127	145	NM	Full/3 months	None
Comont 2014 [29]	France	1	73	M	Concomitant	NM	GM1+	+	Serum+ CSF+	3f	822	101	137	NM	IVIg	Full/2 months	None
Bandyopadhyay 2015 [30]	Japan	1	43	F	14 days	AIDP & AMAN	GM1+	+	Serum+CSF-	3	1950	85.5	350	12	MV/IVIg	Partial/NM	None
Higuchi 2015 [31]	Japan	1	49	M	10 days	AIDP	–	+	Serum+	3	1246	15.4	102	7	IVIg	Full/3 months	None
Perrin 2015 [32]	France	2	Mean: 57	1F, 1 M	Few days	AIDP	NT	+	Serum+(*n* = 1)	3f	Mean:216	Mean:20.6	Mean:173	NM	IVIg	Partial (*n* = 2)/Mean: 9 weeks	None
Fukae 2016 [33]	Japan	3	Mean: 53	3 M	NM	AIDP(*n* = 1) MSF(*n* = 1)	GM1 + (*n* = 1) GQ1b + (*n* = 1)	+	Serum+(*n* = 1)	NT	Mean: 230.3	NM	Mean: 91	Mean:5	IVIg	Full (*n* = 2) partial(*n* = 1)/NM	None
Ji 2016 [34]	South Korea	1	58	M	75 days	NM	NT	+	NT	NT	525	23.59	44.6	0	IVIg	Full/12 months	None
Lei 2017 [35]	China	1	30	M	Concomitant	AIDP	NT	+	NT	NT	4502	83.96	344.19	0	IVIg	Full/3 months	None
Stevens 2017 [36]	Belgium	6	Mean:61	4 M, 2F	NM	AIDP(*n* = 1) AMSAN(*n* = 1) Equivocal(*n* = 1) Demyelinating(*n* = 2) Sensory neuropathy(*n* = 1)	–	+	Serum+(*n* = 2)	NT	Mean:762	Mean:39.27	Mean:69.4	Mean:4.8	IVIg(*n* = 4) PP(*n* = 1) supportive(*n* = 1)	Partial (*n* = 5)/3–6 months Death(*n* = 1)/1 month	NM
Our case	China	1	58	M	11 days	AIDP	–	+	NT	NT	664	51.8	275.3	0	IVIg	Full/6 months	None

*F* female, *M* male, *HEV* hepatitis E virus, *ALT* alanine aminotransferase, + positive, − negative, *CSF* cerebrospinal fluid, *AIDP* acute inflammatory demyelinating polyneuropathy, *AMAN* acute motor axonal neuropathy, *AMSAN* acute motor–sensory axonal neuropathy, *MSF* Miller Fisher syndrome, *NM* not mentioned, *NT* not tested, *MV* mechanical ventilation, *IVIg* intravenous immunoglobulin, *PP* plasmapheresis

Among the 47 cases with available details of nerve conduction studies, 23(48.9%) had experienced AIDP. Other variants of GBS, including AMAN, AMSAN, and sensory neuropathy, were also detected. Anti-ganglioside GM1, anti-ganglioside GM2, and GQ1b were detected in eight patients. Thus, the pathogenesis of GBS-associated HEV may be related to GM1, GM2, and GQ1b antibodies. Among the 31 cases with available details of treatments, 25 used intravenous immunoglobulin (IVIg), 4 used plasmapheresis (PP), and 1 used ribavirin. Some patients recovered spontaneously without IVIg or PP administration. Mechanical ventilation was performed in seven patients due to the involvement of respiratory muscles. Almost all the reviewed patients achieved complete neurological recovery within several weeks to several months, which suggested good prognosis for HEV-associated GBS. However, one patient died after cardiac arrest 1 month after the onset of neurological symptoms.

In conclusion, GBS is an emerging extrahepatic manifestation of HEV infection. HEV infection should be strongly considered in patients with neurological symptoms, especially those with elevated levels of liver enzymes. In our case, HEV infection was confirmed by IgM anti-HEV in the serum. This infection could also be supported by HEV RNA. CSF examination showed an increased level of proteins alongside with pleocytosis, which is supported with the suspicion of GBS. Nevertheless, the elevation of CSF proteins is frequent in encephalitis or encephalopathy as well. Thus, the diagnosis of GBS should be cautious. In removes under other resembling disease’s premise. Testing for HEV genotype and anti-ganglioside antibodies will likely help in further studying the pathogenesis of HEV-associated GBS.

HEV infection is a self-limiting disorder, and most patients require no treatment. IVIg and PP are both effective treatments for GBS. However, IVIg has replaced PP as the first line of treatment in most hospitals due to the convenience and availability of the former [18]. There is no significant difference between intravenous methylprednisolone with IVIg or IVIg alone.

## Additional files

Additional file 1: Table S1. Liver function after admission in our hospital. The patient’s liver function tests showed showed 20 μmol/L total bilirubin, 10 μmol/L conjugated bilirubin, 126 U/L alanine aminotransferase, and 160 U/L gamma-glutamyl transpepidase. (DOCX 16 kb)

Additional file 2: Table S2. Serologic studies for hepatitis virus. Serologic studies for IgM and IgG anti-HEV were both positive. No serological evidence was found for hepatitis A virus, hepatitis B virus, hepatitis C virus, hepatitis D virus. (DOCX 15 kb)

Additional file 3: Table S3. Serological study for HBV, HCV, Syphilis and HIV. Serologic studies for hepatitis B virus, hepatitis C virus, syphilis or human immunodeficiency virus was negative. (DOCX 15 kb)

Additional file 4: Table S4. Serological study for Epstein–Barr virus and cytomegalovirus. Epstein–Barr virus and cytomegalovirus serology indicated positive IgG. (DOCX 14 kb)

Additional file 5: Table S5. The first cerebrospinal fluid (CSF) examination. Cerebrospinal fluid (CSF) examination showed 0/μL monocyte, 4.6 mmol/L glucose level, and 275.3 mg/dL protein level. (DOCX 15 kb)

Additional file 6: Table S6. The second cerebrospinal fluid (CSF) examination. Cerebrospinal fluid (CSF) examination revealed 10/μL monocyte and 85.7 mg/dL protein level. (DOCX 15 kb)

Additional file 7: Table S7. Liver function (one month later after discharge). A month later, the liver function of the patient substantially improved, and his serum levels of AST and ALT were nearly normal. (DOCX 16 kb)

Additional file 8: Table S8. Serological study for HEV(six months later). Six months after discharge, serological study showed IgM anti-HEV antibodies became negative. (DOCX 15 kb)

## Abbreviations

AIDP: Acute inflammatory demyelinating polyneuropathy
ALT: Alanine aminotransferase
AMAN: Acute motor axonal neuropathy
AMSAN: Acute motor-sensory axonal neuropathy
AST: Aspartate aminotransferase
CSF: Cerebrospinal fluid
GBS: Guillain–Barre syndrome
HEV: Hepatitis E virus
IVIg: Intravenous immunoglobulin
MFS: Miller Fisher syndrome
PP: Plasmapheresis

## References

[1] Dalton HR, Bendall R, Ijaz S, Banks M (2008). Hepatitis E: an emerging infection in developed countries. Lancet Infect Dis.

[2] Zhu FC, Zhang J, Zhang XF, Zhou C, Wang ZZ, Huang SJ, Wang H, Yang CL, Jiang HM, Cai JP (2010). Efficacy and safety of a recombinant hepatitis E vaccine in healthy adults: a large-scale, randomised, double-blind placebo-controlled, phase 3 trial. Lancet.

[3] Bazerbachi F, Haffar S (2015). Acute fulminant vs. acute-on-chronic liver failure in hepatitis E: diagnostic implications. Infect Dis (Lond).

[4] Kamar N, Izopet J, Dalton HR (2013). Chronic hepatitis e virus infection and treatment. J Clin Exp Hepatol.

[5] Dalton HR, Kamar N, van Eijk JJ, Mclean BN, Cintas P, Bendall RP, Jacobs BC (2016). Hepatitis E virus and neurological injury. Nat Rev Neurol.

[6] Lee GY, Poovorawan K, Intharasongkroh D, Sa-Nguanmoo P, Vongpunsawad S, Chirathaworn C, Poovorawan Y (2015). Hepatitis E virus infection: epidemiology and treatment implications. World J Virol.

[7] Kamar N, Bendall RP, Peron JM, Cintas P, Prudhomme L, Mansuy JM, Rostaing L, Keane F, Ijaz S, Izopet J (2011). Hepatitis E virus and neurologic disorders. Emerg Infect Dis.

[8] Cheung MC, Maguire J, Carey I, Wendon J, Agarwal K (2012). Review of the neurological manifestations of hepatitis E infection. Ann Hepatol.

[9] Mandal K, Chopra N (2006). Acute transverse myelitis following hepatitis E virus infection. Indian Pediatr.

[10] Bazerbachi F, Haffar S, Garg SK, Lake JR (2016). Extra-hepatic manifestations associated with hepatitis E virus infection: a comprehensive review of the literature. Gastroenterol Rep (Oxf).

[11] Yuki N, Hartung HP (2012). Guillain-Barre syndrome. N Engl J Med.

[12] Kamani P, Baijal R, Amarapurkar D, Gupte P, Patel N, Kumar P, Agal S (2005). Guillain-Barre syndrome associated with acute hepatitis E. Indian J Gastroenterol.

[13] Sood A, Midha V, Sood N (2000). Guillain-Barre syndrome with acute hepatitis E. Am J Gastroenterol.

[14] Loly JP, Rikir E, Seivert M, Legros E, Defrance P, Belaiche J, Moonen G, Delwaide J (2009). Guillain-Barre syndrome following hepatitis E. World J Gastroenterol.

[15] Cronin S, McNicholas R, Kavanagh E, Reid V, O'Rourke K (2011). Anti-glycolipid GM2-positive Guillain-Barre syndrome due to hepatitis E infection. Ir J Med Sci.

[16] Kumar R, Bhoi S, Kumar M, Sharma B, Singh B, Gupta B (2002). Guillain-Barré syndrome and acute hepatitis E: a rare association. JIACM.

[17] Maurissen I, Jeurissen A, Strauven T, Sprengers D, De Schepper B (2012). First case of anti-ganglioside GM1-positive Guillain-Barre syndrome due to hepatitis E virus infection. Infection.

[18] van Doorn PA, Ruts L, Jacobs BC (2008). Clinical features, pathogenesis, and treatment of Guillain-Barre syndrome. Lancet Neurol.

[19] Khanam RA, Faruq MO, Basunia RA, Ahsan AA (2008). Guillain-Barré syndrome associated with acute HEV hepatitis. Med Coll J.

[20] Del Bello A, Arne-Bes MC, Lavayssiere L, Kamar N (2012). Hepatitis E virus-induced severe myositis. J Hepatol.

[21] Tse AC, Cheung RT, Ho SL, Chan KH (2012). Guillain-Barre syndrome associated with acute hepatitis E infection. J Clin Neurosci.

[22] Santos L, Mesquita JR, Rocha PN, Lima-Alves C, Serrao R, Figueiredo P, Reis J, Simoes J, Nascimento M, Sarmento A (2013). Acute hepatitis E complicated by Guillain-Barre syndrome in Portugal, December 2012--a case report. Euro Surveill.

[23] Sharma B, Nagpal K, Bakki SR, Prakash S (2013). Hepatitis E with Gullain-Barre syndrome: still a rare association. J Neuro-Oncol.

[24] Geurtsvankessel CH, Islam Z, Mohammad QD, Jacobs BC, Endtz HP, Osterhaus AD (2013). Hepatitis E and Guillain-Barre syndrome. Clin Infect Dis.

[25] van den Berg B, van der Eijk AA, Pas SD, Hunter JG, Madden RG, Tio-Gillen AP, Dalton HR, Jacobs BC (2014). Guillain-Barre syndrome associated with preceding hepatitis E virus infection. Neurology.

[26] Chen XD, Zhou YT, Zhou JJ, Wang YW, Tong DM (2014). Guillain-Barre syndrome and encephalitis/encephalopathy of a rare case of northern China acute severe hepatitis E infection. Neurol Sci.

[27] Scharn N, Ganzenmueller T, Wenzel JJ, Dengler R, Heim A, Wegner F (2014). Guillain-Barre syndrome associated with autochthonous infection by hepatitis E virus subgenotype 3c. Infection.

[28] Woolson KL, Forbes A, Vine L, Beynon L, McElhinney L, Panayi V, Hunter JG, Madden RG, Glasgow T, Kotecha A (2014). Extra-hepatic manifestations of autochthonous hepatitis E infection. Aliment Pharmacol Ther.

[29] Comont T, Bonnet D, Sigur N, Gerdelat A, Legrand-Abravanel F, Kamar N, Alric L (2014). Acute hepatitis E infection associated with Guillain-Barre syndrome in an immunocompetent patient. Rev Med Interne.

[30] Bandyopadhyay D, Ganesan V, Choudhury C, Kar SS, Karmakar P, Choudhary V, Banerjee P, Bhar D, Hajra A, Layek M (2015). Two uncommon causes of Guillain-Barre syndrome: hepatitis E and Japanese encephalitis. Case Rep Neurol Med.

[31] Higuchi MA, Fukae J, Tsugawa J, Ouma S, Takahashi K, Mishiro S, Tsuboi Y (2015). Dysgeusia in a patient with Guillain-Barre syndrome associated with acute hepatitis E: a case report and literature review. Intern Med.

[32] Perrin HB, Cintas P, Abravanel F, Gerolami R, D'Alteroche L, Raynal JN, Alric L, Dupuis E, Prudhomme L, Vaucher E (2015). Neurologic disorders in Immunocompetent patients with autochthonous acute hepatitis E. Emerg Infect Dis.

[33] Fukae J, Tsugawa J, Ouma S, Umezu T, Kusunoki S, Tsuboi Y (2016). Guillain-Barre and miller fisher syndromes in patients with anti-hepatitis E virus antibody: a hospital-based survey in Japan. Neurol Sci.

[34] Ji SB, Lee SS, Jung HC, Kim HJ, Kim HJ, Kim TH, Jung WT, Lee OJ, Song DH (2016). A Korean patient with Guillain-Barre syndrome following acute hepatitis E whose cholestasis resolved with steroid therapy. Clin Mol Hepatol.

[35] Lei JH, Tian Y, Luo HY, Chen Z, Peng F (2017). Guillain-Barre syndrome following acute co-super-infection of hepatitis E virus and cytomegalovirus in a chronic hepatitis B virus carrier. J Med Virol.

[36] Stevens O, Claeys KG, Poesen K, Saegeman V, Van Damme P (2017). Diagnostic challenges and clinical characteristics of hepatitis E virus-associated Guillain-Barre syndrome. Jama Neurol.

