# Supplementary material for: Guillain-Barre syndrome caused by hepatitis E infection: case report and literature review
Source: BMC Infect Dis. 2018 Jan 23;18:50. doi: 10.1186/s12879-018-2959-2 (PMC5778630; doi:10.1186/s12879-018-2959-2)
Supplement: Supplementary file 1 — Liver function after admission in our hospital. The patient’s liver function tests showed showed 20 μmol/L total bilirubin, 10 μmol/L conjugated bilirubin, 126 U/L alanine aminotransferase, and 160 U/L gamma-glutamyl transpepidase. (DOCX 16 kb) [file 12879_2018_2959_MOESM1_ESM.docx]

Liver function after admission in our hospital

| **Biochemistry test** | | **2015/12/28** | |
| --- | --- | --- | --- |
| **Subject** | **Test result** | **Normal range** | **Unit** |
| Total protein | 71 | 61-83 | g/L |
| Albumin | 37.3 | 35-55 | g/L |
| Globulin | 33.7 | 20-35 | g/L |
| Glutamic-pyruvic transaminase | 126 | 5-40 | U/L |
| Glutamic-oxalacetic transaminease | 29 | 8-40 | U/L |
| Alkaline phosphatase | 150 | 40-150 | U/L |
| Cholinesterase | 6010 | 4500-13000 | U/L |
| Total bile acid | 2 | 1-12 | μmol/L |
| Total bilirubin | 20 | 0-21 | μmol/L |
| Direct bilirubin | 10 | 0-5 | μmol/L |
| Indirect bilirubin | 10 | 3-14 | μmol/L |
| Adenylic deaminase | 10 | 0-18 | U/L |
| Glutamyltranspeptidase | 160 | 11-50 | U/L |
| Glomerular filtration rate | 101.23 |  | mL/min |
| Creatinine | 65 | 59-104 | μmol/L |
| Blood urea nitrogen | 3.7 | 2.9-8.2 | mmol/L |
| Uric acid | 147 | 208-428 | μmol/L |
| Cystain C | 0.59 | 0.4-1.1 | mg/L |
| Triglyceride | 1.22 | 0.3-1.7 | mmol/L |
| Total cholestrol | 3.96 | 3.14-5.86 | mmol/L |
| Potassium | 4.25 | 3.5-5.2 | mmol/L |
| Sodium | 140 | 136-145 | mmol/L |
| Chlorine | 104 | 96-108 | mmol/L |
| Calcium | 2.06 | 2.03-2.54 | mmol/L |
| Phosphorus | 1.4 | 0.87-1.45 | U/L |
| Blood glucose | 4.15 | 3.9-6.1 | U/L |
