# Supplementary material for: Guillain-Barre syndrome caused by hepatitis E infection: case report and literature review
Source: BMC Infect Dis. 2018 Jan 23;18:50. doi: 10.1186/s12879-018-2959-2 (PMC5778630; doi:10.1186/s12879-018-2959-2)
Supplement: Supplementary file 2 — Serologic studies for hepatitis virus. Serologic studies for IgM and IgG anti-HEV were both positive. No serological evidence was found for hepatitis A virus, hepatitis B virus, hepatitis C virus, hepatitis D virus. (DOCX 15 kb) [file 12879_2018_2959_MOESM2_ESM.docx]

Serologic studies for hepatitis virus

| **Antigen&Antibody test of viral hepititis** | | **2015/12/28** | |
| --- | --- | --- | --- |
| **Subject** | **Test result** | **Normal range** | **Unit** |
| HAV-IgM | Nagative | Nagative | / |
| HBsAg | 0.02 | 0-0.05 | IU/mL |
| Anti-HBs | 329.43 | 0-10 | mIU/mL |
| HBeAg | 0.01 | 0-0.18 | PEIU/mL |
| Anti-Hbe | 0.37 | 1-9999 | S/CO |
| HBcAg | 9.61 | 0-1 | S/CO |
| HBV Pre-S1 antigen | Nagative | Nagative | / |
| HCV-Core antigen | Nagative | Nagative | / |
| HCV-IgG | Nagative | Nagative | / |
| HEV-IgG | Positive | Nagative | / |
| HEV-IgM | Positive | Nagative | / |
| HDV antigen | Nagative | Nagative | / |
| HDV-IgG | Nagative | Nagative | / |
| HDV-IgM | Nagative | Nagative | / |
