# Supplementary material for: Guillain-Barre syndrome caused by hepatitis E infection: case report and literature review
Source: BMC Infect Dis. 2018 Jan 23;18:50. doi: 10.1186/s12879-018-2959-2 (PMC5778630; doi:10.1186/s12879-018-2959-2)
Supplement: Supplementary file 3 — Serological study for HBV, HCV, Syphilis and HIV. Serologic studies for hepatitis B virus, hepatitis C virus, syphilis or human immunodeficiency virus was negative. (DOCX 15 kb) [file 12879_2018_2959_MOESM3_ESM.docx]

Serological study for HBV, HCV, Syphilis and HIV

| **Antibody for HBV, HCV, Syphilis and HIV** | | **2015/12/27** | |
| --- | --- | --- | --- |
| **Subject** | **Test result** | **Normal range** | **Unit** |
| HBsAg ELISA | Negative | Negative | / |
| Anti-HIV | Negative | Negative | / |
| HCV-IgG ELISA | Negative | Negative | / |
| Syphilis TPHA | Negative | Negative | / |
