# Supplementary material for: Guillain-Barre syndrome caused by hepatitis E infection: case report and literature review
Source: BMC Infect Dis. 2018 Jan 23;18:50. doi: 10.1186/s12879-018-2959-2 (PMC5778630; doi:10.1186/s12879-018-2959-2)
Supplement: Supplementary file 4 — Serological study for Epstein–Barr virus and cytomegalovirus. Epstein–Barr virus and cytomegalovirus serology indicated positive IgG. (DOCX 14 kb) [file 12879_2018_2959_MOESM4_ESM.docx]

Serological study for Epstein–Barr virus and cytomegalovirus

| **Antibody for Epstein–Barr virus and cytomegalovirus** | | | **2015/12/31** | |
| --- | --- | --- | --- | --- |
| **Subject** | **Test result** | **Normal range** | | **Unit** |
| Cytomegalovirus IgG | 7.8 (Positive) | / | | S/CO |
| Cytomegalovirus IgM | Negative | Negative | | S/CO |
| Epstein–Barr virus-IgG | 5.6 (Positive) | / | | S/CO |
| Epstein–Barr virus-IgM | Negative | Negative | | S/CO |
