# Supplementary material for: Guillain-Barre syndrome caused by hepatitis E infection: case report and literature review
Source: BMC Infect Dis. 2018 Jan 23;18:50. doi: 10.1186/s12879-018-2959-2 (PMC5778630; doi:10.1186/s12879-018-2959-2)
Supplement: Supplementary file 5 — The first cerebrospinal fluid (CSF) examination. Cerebrospinal fluid (CSF) examination showed 0/μL monocyte, 4.6 mmol/L glucose level, and 275.3 mg/dL protein level. (DOCX 15 kb) [file 12879_2018_2959_MOESM5_ESM.docx]

The first cerebrospinal fluid (CSF) examination

| **Examination of cerebrospinal fluid** | | **2015/12/27** | |
| --- | --- | --- | --- |
| **Subject** | **Test result** | **Normal range** | **Unit** |
| Appearance | Colorless transparent liquid | / | / |
| Pandy test | Nagative | Nagative | / |
| Karyocyte | 0 | / | /μL |
| Erythrocyte | 1 | / | /μL |
| Ink stain | Nagative | Nagative | / |
| CSF-Chloridion | 124 | 120-131 | mmol/L |
| CSF-Glucose | 4.6 | 2.5-4.5 | mmol/L |
| CSF-Protein | 2.753 | 0.15-0.45 | g/L |
