# Supplementary material for: Guillain-Barre syndrome caused by hepatitis E infection: case report and literature review
Source: BMC Infect Dis. 2018 Jan 23;18:50. doi: 10.1186/s12879-018-2959-2 (PMC5778630; doi:10.1186/s12879-018-2959-2)
Supplement: Supplementary file 7 — Liver function (one month later after discharge). A month later, the liver function of the patient substantially improved, and his serum levels of AST and ALT were nearly normal. (DOCX 16 kb) [file 12879_2018_2959_MOESM7_ESM.docx]

Liver function (one month later after discharge)

| **Biochemistry test** | | **2016/2/14** | |
| --- | --- | --- | --- |
| **Subject** | **Test result** | **Normal range** | **Unit** |
| Total protein | 68.8 | 61-83 | g/L |
| Albumin | 45.4 | 35-55 | g/L |
| Globulin | 25.4 | 20-35 | g/L |
| Glutamic-pyruvic transaminase | 52 | 5-40 | U/L |
| Glutamic-oxalacetic transaminease | 45 | 8-40 | U/L |
| Alkaline phosphatase | 90 | 40-150 | U/L |
| Cholinesterase | 6930 | 4500-13000 | U/L |
| Total bile acid | 3 | 1-12 | μmol/L |
| Total bilirubin | 11 | 0-21 | μmol/L |
| Direct bilirubin | 4 | 0-5 | μmol/L |
| Indirect bilirubin | 7 | 3-14 | μmol/L |
| Adenylic deaminase | 8 | 0-18 | U/L |
| Glutamyltranspeptidase | 22 | 11-50 | U/L |
| Glomerular filtration rate | 98.67 |  | mL/min |
| Creatinine | 68 | 59-104 | μmol/L |
| Blood urea nitrogen | 5.5 | 2.9-8.2 | mmol/L |
| Uric acid | 264 | 208-428 | μmol/L |
| Triglyceride | 1.6 | 0.3-1.7 | mmol/L |
| Total cholestrol | 4.01 | 3.14-5.86 | mmol/L |
| Potassium | 3.94 | 3.5-5.2 | mmol/L |
| Sodium | 144 | 136-145 | mmol/L |
| Chlorine | 106 | 96-108 | mmol/L |
| Calcium | 2.22 | 2.03-2.54 | mmol/L |
| Phosphorus | 0.99 | 0.87-1.45 | U/L |
| Blood glucose | 5.09 | 3.9-6.1 | U/L |
