# Supplementary material for: Guillain-Barre syndrome caused by hepatitis E infection: case report and literature review
Source: BMC Infect Dis. 2018 Jan 23;18:50. doi: 10.1186/s12879-018-2959-2 (PMC5778630; doi:10.1186/s12879-018-2959-2)
Supplement: Supplementary file 8 — Serological study for HEV(six months later). Six months after discharge, serological study showed IgM anti-HEV antibodies became negative. (DOCX 15 kb) [file 12879_2018_2959_MOESM8_ESM.docx]

Serological study for HEV(six months later)

| **Antibodys for HEV** | | **2016/8/15** | |
| --- | --- | --- | --- |
| **Subject** | **Test result** | **Normal range** | **Unit** |
| HEV-IgM | Negative | Negative | / |
| HEV-IgG | Positive | Negative | / |
